# Molecular detection of *Theileria* spp. in native sheep and estimation of hemato-biochemical parameters from Sulaimani province/Iraq

**DOI:** 10.3389/fvets.2022.1059599

**Published:** 2022-12-16

**Authors:** Shadan Hassan Abdullah, Hiewa Othman Dyary, Nahla Mohammed Saeed

**Affiliations:** ^1^Department of Microbiology, College of Veterinary Medicine, University of Sulaimani, Sulaymaniyah, Iraq; ^2^Department of Basic Sciences, College of Veterinary Medicine, University of Sulaimani, Sulaymaniyah, Iraq

**Keywords:** *Theileria*, sheep, blood, PCR, Sulaimani

## Abstract

Theileriosis, the hemoprotozoan infection, is an endemic condition in tropical and subtropical areas. In this study, conventional PCR analysis was applied to detect the natural infection of native sheep with theileriosis and estimate its effect on hemato-biochemical parameters. The study was carried out in five regions of Sulaimani province, northern Iraq. From May to October 2021, a total of 360 blood samples were collected randomly from the jugular vein of sheep belonging to 23 flocks with a history of tick infestations. After PCR for theileriosis, the hematobiochemical parameters were evaluated by an automatic analyzer using commercial kits. The PCR results represented that 71.7% of the examined sheep were infected with *Theileria* parasites. The highest prevalence rate (74.6%) was reported in Said Sadiq, and the lowest prevalence (69.5%) was from Bazian. The infection rates in Mawat, Qaradagh, and Sharazoor were 73.1, 70.3, and 71.8%, respectively. The hemogram data revealed a significant decrease in erythrocyte count, hemoglobin concentration, and hematocrit values. Erythrocyte indices also showed significant increases in MC, MCH, and MCHC levels, but no significant differences were detected between the counting of leukocytes, lymphocytes, and granulocytes. Biochemical analysis revealed a significant decrease in total protein, albumin, and creatinine levels with a significant increase in urea and AST levels in infected sheep with theileriosis. Alteration in hemato-biochemical parameters from infected animals can outline the impact of theileriosis on body metabolism and blood factors in naturally infected sheep.

## Introduction

Theileriosis is a hemoprotozoan disease caused by Apicomplexan parasites of the genus *Theileria*. Infection with *Theileria* spp. is considered an important devastating disease of small ruminants, appearing in subacute, acute, or chronic forms (1). Theileriosis is associated with substantial economic losses in livestock due to high morbidity and mortality, especially in tropical and subtropical areas (2, 3). Several species cause theileriosis in ovines with various pathogenicities. *T. lestoquardi, T. luwenshuni*, and *T. uilenbergi* produce malignant infections, while *T. ovis, T. separata*, and *T. recondita* produce mild-type infections. Also, natural infection with *T. annulata* in sheep has been reported worldwide (4). Other species of sheep have also been reported, including *Theileria* sp. OT1, *Theileria* sp. OT3, and *Theileria* sp. MK (5, 6). The parasite was transmitted through vector ticks belonging to the genera *Rhipicephalus, Hyalomma*, and *Haemaphysalis* (7). As a common vector-borne livestock infection, *Theileria* infects leukocytes and erythrocytes (8).

Theileriosis occurs after establishing schizont stages within the cytoplasm of susceptible host lymphocytes, with subsequent development of merozoites invading the erythrocytes (9). Other stages of the parasite's life cycle occur in the tick's gut, leading to the development of infective sporozoites (10). Previous studies represented that various factors play a role in the incidence and dissemination of ovine theileriosis (11, 12). The main dispersing factor associated with the prevalence of theileriosis is the existence of susceptible hosts and ectoparasitic vectors (13, 14). The dormant stage of infection is vital in disease epidemiology because chronic carriers act as reservoirs for tick-borne infection. Molecular techniques enable the sensitive and specific detection of pathogens (6). Furthermore, changes in blood biomarker profiles can provide valuable information on the severity of the infection and help measure the prognosis of the disease and determine the applied therapy (15, 16).

Blood and biochemical profiles, including total proteins, globulins, albumin, glucose, bilirubin, cholesterol, urea, and creatinine, can be helpful in disease diagnosis, and serum levels of ALT and AST enzymes give essential evidence about the health status of muscular and liver tissues. These hematobiochemical data effectively distinguish between healthy and diseased animals in veterinary studies (14). Hematological parameters, such as total erythrocyte count, hematocrit, hemoglobin concentration, mean corpuscular volume, mean corpuscular hemoglobin concentration, and total and differential leukocyte count, provide vital information on the health status of the animal (17).

Detection of ovine theileriosis and verification have been performed adequately through different diagnostic methods, including PCR and sequencing of amplified DNA products. In contrast, there was little about its effects on hemato-biochemical parameters. Accordingly, the study was designed to define the impact of natural *Theileria* infection on body metabolism and blood factors in naturally infected sheep.

## Materials and methods

### Study areas and sampling

**Figure 1:**
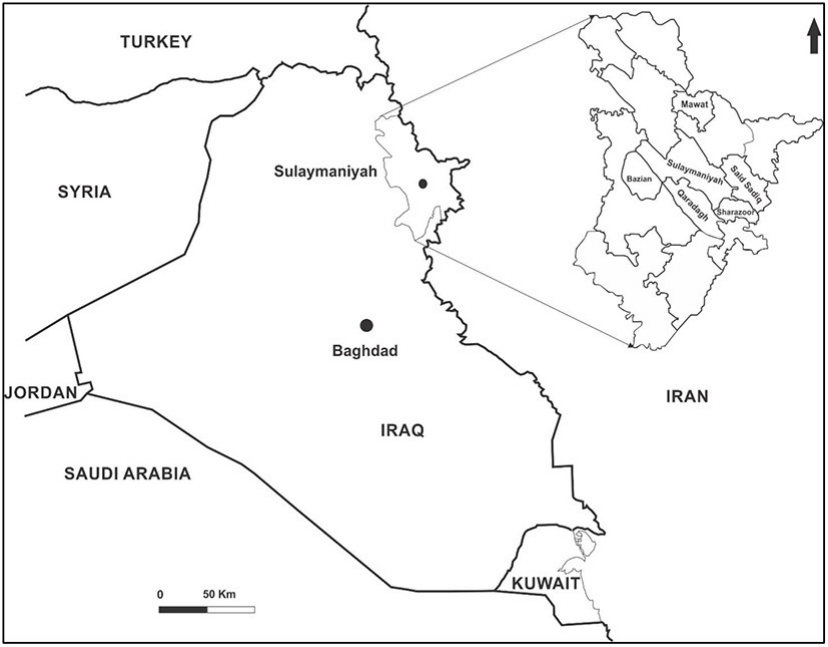
Iraqi map showing the study areas in Sulaimani.

The study was carried out in the Sulaimani province and its surrounding districts in the northern region of Iraq, namely, Bazian, Mawat, Qaradagh, Said Sadiq, and Sharazoor (Figure 1). The number of sheep in the included districts was estimated at 60,000 sheep. From May to October 2021, blood samples were randomly collected from 360 native sheep within 23 herds with a history of tick infestations. The sample size accounted for about 0.6% of the sheep in the study area, calculated based on a 95% confidence interval and a 5% error margin. The age of the included animals was more than 1 year, and of both sexes. About 7 mL of blood was drawn aseptically from the jugular vein. Two mL of blood were placed in an anticoagulant tube (EDTA) for hematological evaluation and molecular detection, and the rest in free anticoagulant tubes for serum collection. The samples were kept at a low temperature until transported to the laboratory, then processed, and the obtained sera were collected in Eppendorf and stored at −20°C until used for biochemical analysis. After hematological estimation, the remaining blood samples were stored similarly in a deep freezer for DNA extraction.

### Laboratory examination

#### Molecular study

The genomic DNA was extracted from whole blood samples using a DNA extraction kit from GeNet Bio (South Korea). Following the manufacturer's instructions, the extracted DNA concentration (ng/mL) was measured using a NanoDrop spectrophotometer and stored at −20°C until used. By applying a conventional PCR assay, the *Theileria* parasite was detected targeting the 18S rRNA gene, using a previously designed specific oligonucleotide primer set with a 1098 bp portion of the gene (F: 5′-AGTTTCTGACCTATCAG-3′ and R: 5′-TTGCCTTAAACTTCCTTG-3′) described by Allsopp et al. (18).

For the PCR assay, the GeNet Bio green master mix from South Korea was used to screen all extracted DNA samples in a total volume of 20 μL. Amplification was achieved in a programmable thermocycler (Prime, UK) with the reactions starting with predenaturation at 94°C for 5 min, followed by 35 cycles of denaturation at 94°C for 1 min, annealing at 60°C for 1 min, and extension at 72°C for 1 min. The final extension was carried out at 72°C for 7 min, then ended by cooling at 4°C. After amplification, 7 μL of PCR products were stained with ethidium bromide and run on a 1.5% agarose gel in TBE buffer for 80 min with 200 mA and 90 volts. After that, the specific band was visualized using a UV illuminator. The amplified positive samples were compared to the 1098 bp of the 18S rRNA gene.

#### Hematological studies

The hematological parameters studied included total erythrocyte count (millions/μL), hemoglobin concentration (Hb, g/dL), and hematocrit value (HCT, %). Several erythrocytic indices were also estimated, including Mean Corpuscular Volume (MCV), Mean Corpuscular Hemoglobin (MCH), Mean Corpuscular Hemoglobin Concentration (MCHC), Total Leukocytes Counts (TLC), Lymphocytes (LYM), and Granulocytes (GRA) using a hematology analyzer for infected and healthy animals (19).

#### Biochemical study

Sera from infected and uninfected animals were analyzed for biochemical parameters *via* an auto-analyzer (Alpha Classic, Sweden). The studied biochemical profiles, including total serum protein (TSP), serum albumin (SA), blood urea nitrogen (BUN), serum creatinine (CRE), and Aspartate Transaminase (AST), were estimated through spectrophotometry using commercial test kits (20).

### Statistical analysis

Data analysis was performed through the Statistical Package for Social Sciences (SPSS version 24.0, IBM, USA). A comparison of the hematobiochemical parameters between infected and uninfected sheep was made using an independent samples *t*-test. A probability level of <0.05 was considered significantly different.

## Results

**Figure 2:**
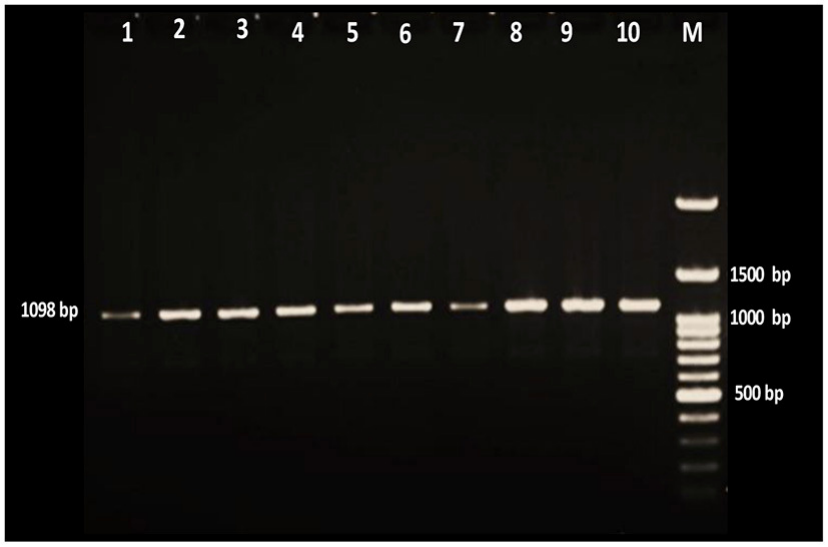
Gel electrophoresis of amplified *Theileria* positive products with ethidium bromide stain. Lane M is a 100-bp DNA ladder, and lanes 1–10 were positive samples for the *Theileria* parasite.

The data of the present study revealed that of 360 native sheep examined, 71.7% were positive for *Theileria* protozoa. The PCR-based diagnosis of *Theileria* revealed the highest prevalence rate in Said Sadiq (74.6%) and the lowest prevalence in Bazian (69.5%), as shown in Figure 2 and Table 1.

**Table 1:** Occurrence of *Theileria* spp. in sheep according to regions of the Sulaymaniyah province.

**Location**	**Examined samples**	**Number infected**	**Prevalence (%)**
Bazian	82	57	69.5
Mawat	67	49	73.1
Qaradagh	74	52	70.3
Said Sadiq	59	44	74.6
Sharazoor	78	56	71.8
Total	360	258	71.7

The hematological profiles of infected sheep represented a significant decrease in total erythrocyte count, hemoglobin, and hematocrit. Furthermore, the erythrocytic indices of MCV, MCH, and MCHC recorded significantly higher values. Furthermore, a decrease in total leukocytes, lymphocytes, and granulocytes was nonsignificant (*P* > 0.05).

Serum concentrations of total protein, albumin, and creatinine decreased significantly in infected sheep. On the contrary, significant increases in AST and urea concentrations were recorded in animals infected with *Theileria* (Table 2).

**Table 2:** Hematological and biochemical parameters of infected and uninfected sheep with *Theileria* spp.

**Parameter**	**Infected sheep (258)**	**Uninfected sheep (102)**	***P*-value**
Erythrocytes	7.85 ± 0.04	8.66 ± 0.07	0.000
Hemoglobin	9.46 ± 0.05	9.90 ± 0.01	0.000
Hematocrit	27.86 ± 0.16	29.65 ± 0.30	0.000
MCV	35.49 ± 0.10	34.23 ± 0.17	0.000
MCH	12.04 ± 0.03	11.43 ± 0.06	0.000
MCHC	33.94 ± 0.03	33.40 ± 0.03	0.000
Leukocytes	10.70 ± 0.24	10.55 ± 0.37	0.747
Lymphocytes	5.52 ± 0.15	5.62 ± 0.25	0.738
Granulocytes	2.60 ± 0.16	2.68 ± 0.28	0.800
Total Proteins	6.22 ± 0.03	7.43 ± 0.06	0.000
Albumin	3.15 ± 0.02	3.76 ± 0.03	0.000
AST	110.91 ± 1.58	79.47 ± 1.38	0.000
Creatinine	0.98 ± 0.01	1.26 ± 0.02	0.000
Urea	34.43 ± 0.58	21.43 ± 0.62	0.000

Values are presented as means ± SEM. MCV, mean corpuscular volume; MCH, Mean corpuscular hemoglobin; MCHC, Mean corpuscular hemoglobin concertation; AST, Aspartate transaminase. Statistical analysis = independent samples t-test.

## Discussion

Ovine theileriosis represents the endemic condition in the study area with a significant impact on the livestock industry. The present study showed that the prevalence of theileriosis in native sheep was 71.7%, in agreement with the study data reported by Abdullah and Ali (6) 5 years earlier, with a prevalence rate of 76.4% in the Sulaimani province. A lower prevalence than the current study has been reported in other locations. Infection rates of 62.5% in Iran, 54.4% in Oman, and 53.0% in Pakistan were found by Zarei et al. (21), Al-Hamidhi et al. (17), and Shahzad et al. (22), respectively. In other studies, Nangru et al. (23) and Riaz and Tasawar (14) reported 44% in India and 39.5% in Pakistan.

In the current study, samples were collected from small ruminant flukes with a history of tick infestation. Such a high prevalence rate indicated that tick infestation is a chief factor for the persistence of theileriosis in flukes. The significantly higher prevalence of theileriosis in tick-infested animals compared to tick-free sheep was reported by Ullah et al. (24). The highest prevalence of *Theileria* spp. was recognized in Said Sadiq, while the lowest was in Bazian based on PCR. The results indicated different prevalence rates of ovine theileriosis at different sampling sites. Different factors such as husbandry practices, nutritional deficiencies, breed, sex, flock size, climatic conditions (humidity, temperature), and contagious infections directly affect health status. Sheep infections with *Theileria* spp. are associated with the intra-leukocyte schizont and intra-erythrocyte piroplasm stages, which affect the hemato-biochemical changes, and the severity of these changes is related to the virulence of the parasite strain, the infectious dose, the animal breed, and immune status (25).

The present study results show that *Theileria* infection in ovine led to alterations in hemato-biochemical parameters. The hemogram showed a significant decrease in total erythrocyte count, hemoglobin concentration, and hematocrit value. Furthermore, significantly higher values of the MCV, MCH, and MCHC were recorded from PCR-positive samples. These findings agreed with previous studies (24, 26). Infection with *Theileria* spp. results in increased oxidative stress in infected animals, leading to increased fragility of erythrocytes due to membrane lysis and a lower concentration of hemoglobin (27). The reduction of erythrocyte counts might be associated with phagocytosis of the infected cells by an endothelial system of the spleen, lymph nodes, and other organs (28). The total leukocytes, lymphocytes, and granulocyte counts did not differ between the infected and uninfected sheep. Al-Hamidhi, Elshafie, Yaghfoori, Morrison, Johnson and Babiker (17) observed the same finding, while Shahzad (21) reported a significant decrease in WBCs, monocytes, and lymphocytes. A decrease in total cell count is presented by clinical signs like hemolytic anemia, dyspnea, and lethargy (27).

The biochemical analysis showed significantly lower levels of total protein, albumin, and creatinine concentrations with a significant increase in blood urea levels and serum AST enzyme activities of animals infected with *Theileria*. In agreement with the study findings (16, 22), also recognized that *Theileria* parasites cause a significant decrease in protein concentration. The reduction in serum total protein content is due to hypoalbuminemia caused by decreased albumin synthesis, which is the direct or indirect effect of the parasite on hepatocytes, leading to liver failure. Decreased dietary protein intake and diarrhea have also been related factors. Hypoproteinemia and hypoalbuminemia might also result from the accumulation of extravascular proteinaceous fluid after the lymph nodes are affected (29).

A significant decrease in serum creatinine concentration in infected animals was consistent with previous reports by Shahzad et al. (22) in sheep infected with theileriosis. In contrast (30), reported a nonsignificant decrease in creatinine concentration from infected sheep with theileriosis. Furthermore, current data contradict what was reported by Col and Uslu (16) and (31), with a reverse pattern of a nonsignificant increase in serum creatinine levels in cattle and sheep. The concentration of blood urea in the current study was significantly increased, following the findings of Baghshani et al. (30) and Al-Fetly (31). However, Riaz and Tasawar (14) have reported a nonsignificant increase in urea levels among infected sheep. Increased urea concentrations in animals infected with *Theileria* could be related to kidney damage (16) and strongly associated with the level of parasitemia (14).

The measurement of enzyme concentration is another laboratory trial for disease evaluation. An increase in serum enzyme activity indicates the occurrence of necrosis or destruction in the liver or muscle tissues (32). Detection of AST and ALT serum concentrations provides a significant indication for determining the physiological function of the liver. An increase in the level of AST concentration was significant in *Theileria-*infected sheep compared with uninfected. Other researchers have reported similar findings (19, 30, 32). This rise in AST levels might be associated with hepatic dysfunction or muscular distress (16). Furthermore, the toxic metabolites of *Theileria* spp. have harmful effects on liver cells, which could raise the level of enzymes (33).

## Conclusions

The study data denoted a high prevalence of *Theileria* infection among sheep, confirming the endemicity of the disease in the studied area. *Theileria* spp. are dominant hemoparasites in the study area, confirming the high population of transmitter vector ticks for different *Theileria* spp. Under traditional husbandry conditions, subclinically infected animals remain a source of tick infection. The annual costs of animal loss and treatment should be considered. Alteration in some hematological and biochemical profiles of infected sheep reveals the influence of theileriosis on metabolic disturbances due to its harmful effects on the body's organs and systems. Hence, a reduction in animal production might result from chronic infection with theileriosis.

## Data availability statement

The original contributions presented in the study are included in the article/supplementary material, further inquiries can be directed to the corresponding author.

## Ethics statement

The animal study was reviewed and approved by Animal Care and Use Committee at the College of Veterinary Medicine, University of Sulaimani. Written informed consent was obtained from the owners for the participation of their animals in this study.

## Author contributions

SA conducted the design, methodology, and writing of the manuscript. HD and NS participated in the data interpretation and editing of the manuscript. All authors contributed to the article and approved the submitted version.
